# PredDBP-Stack: Prediction of DNA-Binding Proteins from HMM Profiles using a Stacked Ensemble Method

**DOI:** 10.1155/2020/7297631

**Published:** 2020-04-13

**Authors:** Jun Wang, Huiwen Zheng, Yang Yang, Wanyue Xiao, Taigang Liu

**Affiliations:** ^1^College of Information, Shanghai Ocean University, Shanghai 201306, China; ^2^School of Engineering, University of Melbourne, Victoria 3010, Australia; ^3^School of Information Management, Nanjing University, Nanjing 210023, China; ^4^School of Information, Syracuse University, Syracuse, NY 13244, USA

## Abstract

DNA-binding proteins (DBPs) play vital roles in all aspects of genetic activities. However, the identification of DBPs by using wet-lab experimental approaches is often time-consuming and laborious. In this study, we develop a novel computational method, called PredDBP-Stack, to predict DBPs solely based on protein sequences. First, amino acid composition (AAC) and transition probability composition (TPC) extracted from the hidden markov model (HMM) profile are adopted to represent a protein. Next, we establish a stacked ensemble model to identify DBPs, which involves two stages of learning. In the first stage, the four base classifiers are trained with the features of HMM-based compositions. In the second stage, the prediction probabilities of these base classifiers are used as inputs to the meta-classifier to perform the final prediction of DBPs. Based on the PDB1075 benchmark dataset, we conduct a jackknife cross validation with the proposed PredDBP-Stack predictor and obtain a balanced sensitivity and specificity of 92.47% and 92.36%, respectively. This outcome outperforms most of the existing classifiers. Furthermore, our method also achieves superior performance and model robustness on the PDB186 independent dataset. This demonstrates that the PredDBP-Stack is an effective classifier for accurately identifying DBPs based on protein sequence information alone.

## 1. Introduction

DNA-binding proteins (DBPs) are fundamental in the process of composing DNA and regulating genes. They execute intercellular and intracellular functions such as transcription, DNA replication, recombination, modification, and other biological activities associated with DNA [1]. As the significant role of DBPs undertaken, it has become one of the hot research topics to effectively identify DBPs in the field of protein science. The past decade has witnessed tremendous progress in the DBP recognition, including experimental methods, and computational methods [2]. In the early researches, DBPs were detected by laborious experimental techniques such as filter binding assays, genetic analysis, X-ray crystallography, chromatin immune precipitation on microarrays, and nuclear magnetic resonance [3]. With the rapid development of high-throughput sequencing technology and growing extension of protein sequence data, more efficient and accurate machine learning (ML) methods are implemented and applied for the classification of DBPs [4, 5].

Feature encoding schemes and classification algorithms have great impacts on the performance of ML-based methods. Feature representation numerically formulates diverse-length protein sequences as fixed-length feature vectors, which could be categorized into structure-based models and sequence-based models. Structure-based methods rely on the structure information of proteins such as the spatial distribution, net charge, electrostatic potential, the dipole moment, and quadrupole moment tensors [6, 7]. However, the great difficulty of acquiring the high-resolution crystal structure of proteins and the insufficient quantity of proteins with known structure information heavily limit the use of structure-based predictors [8].

In contrast, the sequence-based methods have become more popular since sequence features are usually easier to extract and more convenient to use. These sequence-based features of proteins are classified into three types: (1) composition-based features, such as amino acid composition (AAC) [9], dipeptide composition [10], and pseudo AAC [11–13]; (2) autocorrelation-based features, including autocross covariance [14, 15], normalized Moreau-Broto autocorrelation [8], and physicochemical distance transformation [16]; and (3) profile-based features, including position-specific score matrix (PSSM) [17–19] and hidden markov model (HMM) [20]. Generally, autocorrelation-based features perform better than composition-based features, and profile-based features outperform autocorrelation-based features [21].

Previous studies have demonstrated the importance of PSSM-based features for enhancing DBPs prediction. For example, Kumar et al. initially adopted evolutionary information embedded in the PSSM profile to identify DBPs and achieved a well-performed result [17]. Waris et al. produced an ingenious classifier by integrating the PSSM profile with dipeptide composition and split AAC [18]. Zou et al. proposed a fuzzy kernel ridge regression model to predict DBPs based on multiview sequence features [22]. Ali et al. introduced the DP-BINDER model for the discrimination of DBPs by fusing physicochemical information and PSSM-based features [23]. In the recent study, Zaman et al. built an HMMBinder predictor for the DBP recognition problem by extracting monogram and bigram features derived from the HMM profile [20]. They also experimentally proved that the HMM-based features are more effective for the prediction of DBPs than the PSSM-based features, especially on the jackknife test. Nevertheless, HMMBinder achieved relatively poor performance on the independent test. Accordingly, there is still more scope to improve the DBP prediction by exploring highly recognizable features from the HMM profile.

Prediction of DBPs is usually formulated as a supervised learning problem. In recent years, many classification algorithms have been adopted to solve this problem, including support vector machine (SVM) [24–26], random forest (RF) [27, 28], naive Bayes classifier [3], ensemble classifiers [29–31], and deep learning [32–34]. Among these models, stacked generalization (or stacking) is an ensemble learning technique that takes the outputs of base classifiers as input and attempts to find the optimal combination of the base learners to make a better prediction [35]. Xiong et al. constructed a stacked ensemble model to predict bacterial type IV secreted effectors from protein sequences by using the PSSM-composition features [36]. Recently, Mishra et al. developed a StackDPPred method for the effective prediction of DBPs, which utilized a stacking-based ML method and features extracted from the PSSM profiles [29].

Inspired by the work of Zaman and Mishra, respectively, we propose a stacked ensemble method, called PredDBP-Stack, to further improve the performance of DBP prediction by exploring valuable features from the HMM profiles. First, we convert the HMM profiles into 420-dimensional feature vectors by fusing AAC and transition probability composition (TPC) features. Next, six types of ML algorithms are adopted to implement base classifiers in the first stage. Then, the optimal combination of base learners is searched, and the prediction probabilities of these selected base learners are used as inputs to the meta-classifier to make the final prediction in the second stage. Compared with existing state-of-the-art predictors, our method performs better on the jackknife cross validation as well as on the independent test.

## 2. Materials and Methods

**Figure 1:**
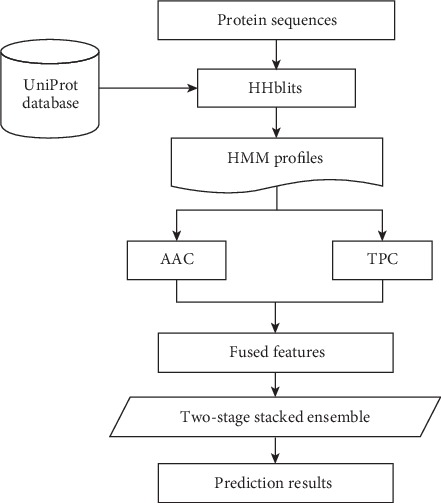
System diagram of PredDBP-Stack.

In this section, we describe all details about the proposed prediction model for identifying DBPs. The system diagram of the PredDBP-Stack methodology is illustrated in Figure 1. Several major intermediate steps in the development process of PredDBP-Stack are specified in the following subsections.

### 2.1. Datasets

The construction of a high-quality benchmark dataset is crucial for building a robust and reliable ML-based predictive model. In this study, two well-established datasets, i.e., PDB1075 [5] and PDB186 [3], are adopted to examine the performance of our predictor. The PDB1075 dataset consists of 1075 protein sequences with 525 DBPs and 550 non-DBPs, which are applied for model training and testing by using the jackknife cross validation. The PDB186 dataset is designed as an independent test dataset that contains 93 DBPs and 93 non-DBPs. All protein sequences in these two datasets were downloaded from the Protein Data Bank [37] and have been filtered rigorously by removing those with relatively high similarity (≥25%) or those with too small length (<50 amino acids) or involving unknown residues such as “X”.

### 2.2. Feature Extraction

#### 2.2.1. HMM Profiles

HMM profiles are supposed to contain rich evolution information of the query proteins and have been widely used in bioinformatics, such as protein remote homology detection [38], DBP prediction [20], and protein fold recognition [39]. In this study, HMM profiles are generated from the multiple sequence alignments by running four iterations of the HHblits program [40] against the latest UniProt database [41] with default parameters. Similar to PSSM profile, we only use the first 20 columns of the HMM profile in the form of an *L* × 20 matrix where *L* represents the length of the query protein sequence. Each element from the HMM profile is normalized by using the following function:

(1) $$\begin{array}{c} f\left(x\right)=\left\{\begin{array}{cc} 0, & \text{if }x=*,\\ \;2^{-x/1000}, & \text{else}, \end{array}\right. \end{array}$$

where *x* is the original value of the HMM profile.

#### 2.2.2. Feature Extraction from HMM Profiles

Feature extraction often plays an important role in most protein classification problems, which has a direct impact on the prediction accuracy of ML-based predictors. In this study, a simple and powerful feature encoding scheme by extracting AAC and TPC features is adopted to convert the HMM profiles into fixed-length feature vectors.

Since DNA-binding preference of a protein is closely related to its AAC [9], we first obtain AAC features from the HMM profile by using the following formula:

(2) $$\begin{array}{c} x_{j}=\frac{1}{L}\overset{L}{\underset{i=1}{\sum }}h_{i,j}\left(j=1,2,\cdots ,20\right), \end{array}$$

where *h*_*i*,*j*_ is the value in the *i*^th^ row and *j*^th^ column of the HMM profile. *x*_*j*_ (1 ≤ *j* ≤ 20) is the composition of amino acid type *j* in the HMM profile and represents the average score of the amino acid residues in the query protein being changed to amino acid type *j* during the evolution process. AAC based on the HMM profile is a simple and intuitive feature; however, it ignores the role of sequence-order information.

To partially reflect the local sequence-order effect, TPC features are computed from the HMM profile as follows:

(3) $$\begin{array}{c} y_{i,j}=\frac{\sum_{k=1}^{L-1}h_{k,i}\times h_{k+1,j}}{\sum_{j=1}^{20}\sum_{k=1}^{L-1}h_{k,i}\times h_{k+1,j}}\;\left(1\leq i,j\leq 20\right). \end{array}$$

To include evolution information and sequence-order information, a 420-dimensional vector is finally employed to represent a protein by fusing AAC and TPC features. We call this feature encoding method AATP-HMM in this study.

### 2.3. Classification Algorithm

**Figure 2:**
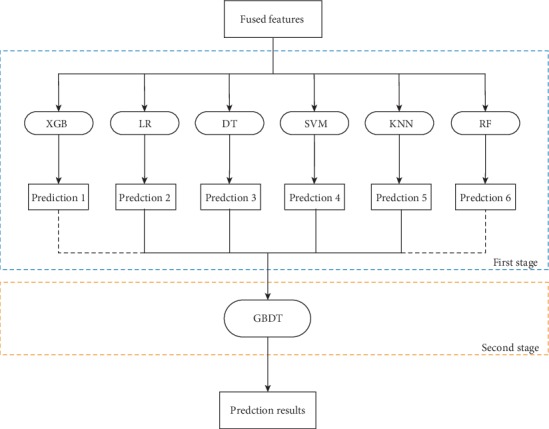
The framework of a two-stage stacked ensemble scheme.

In this study, we apply one of the effective ensemble techniques called stacking [35] to achieve the performance improvement of the DBP predictor. Stacking makes up the limitation of the single classifier by integrating prediction results from multiple classification algorithms. There are two stages in our stacked ensemble scheme (Figure 2). For the first stage, various classification algorithms are employed individually as base classifiers to produce prediction class probabilities. For the second stage, these probabilities as inputs are taken into the meta-classifier in different combinations to generate desired prediction results.

To construct the well-behaved stacked model (SM) with the optimal combination of base classifiers, we explore six classification algorithms: (i) SVM with radial basis kernel function (RBF) [42], (ii) K Nearest Neighbor (KNN) [43], (iii) Logistic Regression (LR) [44], (iv) RF [45], (v) Decision Tree (DT) [46], and (vi) extreme Gradient Boosting (XGB) [47]. All of these algorithms are implemented by using scikit-learn library [48] in Python with the ideal parameters tuned based on the grid search strategy.

Taking into account the underlying principle of each classification algorithm and their prediction performance, we select three top learners, i.e., SVM (RBF), RF, and XGB, to, respectively, combine with other base classifiers. Also, we build the SM with these three best-performed classifiers and the one with all classification models. The following SMs are five combinations of base classifiers in this study:

(i) SM1: KNN, LR, DT, SVM (RBF)

(ii) SM2: KNN, LR, DT, XGB

(iii) SM3: KNN, LR, DT, RF

(iv) SM4: SVM (RBF), XGB, RF, and

(v) SM5: KNN, LR, DT, SVM (RBF), RF, XGB

In our stacked ensemble scheme, we adopt Gradient Boosting Decision Tree (GBDT) [49] as the meta-classifier to perform the final prediction of DBPs. Gradient boosting is a powerful ML technique, which produces a prediction model in the form of an ensemble of weak learners, typically DT [50]. Due to the arbitrary of choosing the loss function, GBDT could be customized to any particular ML task.

### 2.4. Performance Evaluation

To evaluate the performance of PredDBP-Stack, we first implement the jackknife cross-validation test on the PDB1075 dataset. In the jackknife test, every protein is tested one by one by the predictor trained with the remaining proteins in the benchmark dataset. Next, the independent test on the PDB186 dataset is also performed to examine the generalization ability of the proposed model. In this study, four widely used performance metrics are employed to compare PredDBP-Stack with several state-of-the-art models for identifying DBPs, including Overall Accuracy (OA), Sensitivity (SN), Specificity (SP), and Matthew's correlation coefficient (MCC) [51–54]. These metrics are formulated as follows:

(4) $$\begin{array}{c} \text{OA}=\frac{\text{TP}+\text{TN}}{\text{TP}+\text{FP}+\text{TN}+\text{FN}}, \end{array}$$

(5) $$\begin{array}{c} \text{SN}=\frac{\text{TP}}{\text{TP}+\text{FN}}, \end{array}$$

(6) $$\begin{array}{c} \text{SP}=\frac{\text{TN}}{\text{TN}+\text{FP}}, \end{array}$$

(7) $$\begin{array}{c} \text{MCC}=\frac{\text{TP}\times \text{TN}-\text{FP}\times \text{FN}}{\sqrt{\left(\text{TP}+\text{FP}\right)\times \left(\text{TP}+\text{FN}\right)\times \left(\text{TN}+\text{FP}\right)\times \left(\text{TN}+\text{FN}\right)}}, \end{array}$$

where TN, FN, TP, and FP indicate the number of true negative, false negative, true positive, and false positive samples, respectively. Additionally, the area under the Receiver Operating Characteristic (ROC) Curve (AUC) is also computed as it is a powerful metric for evaluating the performance of a binary predictor. The larger the AUC value, the better the performance of the model.

## 3. Results and Discussion

### 3.1. Performance of Base Classifiers

Based on the AATP-HMM feature representation, we first analyze the predictive power of six classifiers, i.e., DT, KNN, LR, XGB, RF, and SVM employed in the base level of stacking. The models are tested on the PDB1075 dataset by using the jackknife cross validation and experimental results are shown in Table 1.

**Figure 3:**
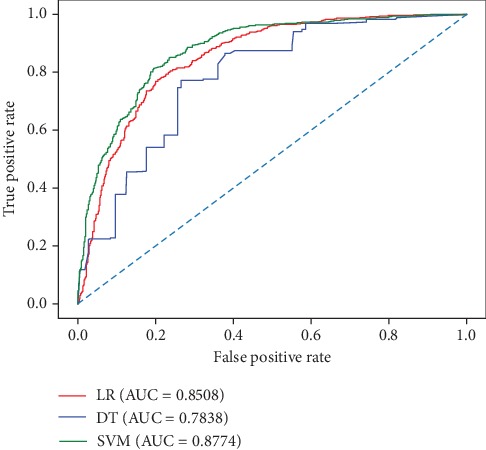
ROC curves of LR, DT, and SVM classifiers on the PDB1075 dataset using the jackknife test.

Table 1 indicates that the optimized SVM with RBF-kernel provides the highest performance in terms of OA, MCC, and AUC compared to the other methods for the prediction of DBPs. Moreover, the RF method obtains the best SN value of 83.4%, and the XGB method gives an outstanding SP value of 80.69%. It is also evident that the DT model performs worst in this task. In addition, the algorithms of KNN and LR show the acceptable performance with the AUC value larger than 0.8. To assure the distinct and high quality of the target figure, only three ROC curves corresponding with LR, DT, and SVM models are shown in Figure 3, which illustrates the consistent findings with Table 1.

### 3.2. Performance of Meta-Classifiers

To find out the optimal combination of base learners, we construct five SMs with different classifiers as follows. As SVM, XGB, and RF are the top three competitive classifiers in the above tests; each one of them is combined with the remaining classifiers to formulate an SM, namely SM1, SM2, and SM3, respectively. The combination of the three outstanding classifiers and all classifiers are formulated as SM4 and SM5. For all the SMs, the meta-classifier in the second stage is GBDT. The performance of five SMs on the PDB1075 dataset using the jackknife test is shown in Table 2.

**Figure 4:**
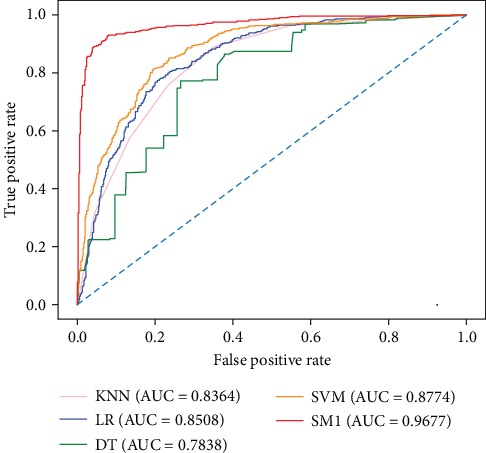
ROC curves of SM1 and its base classifiers on the PDB1075 dataset.

From Table 2, we observe that SM1, SM2, SM3, and SM5 provide similar performance with the OA larger than 90%. However, SM4 produces less competitive scores on the five evaluation measures. It may imply that the combination of the top three competitive classifiers does not mean an advantageous result. Additionally, SM1, which employs KNN, LR, DT, and SVM (RBF) as base learners and GBDT as a meta-classifier, achieves the highest scores on the OA, SN, MCC, and AUC, respectively. SM2 gives the best SP of 92.55%. We also plot the ROC curves for SM1 and its four base classifiers in Figure 4, which demonstrates that stacked generalization can indeed improve the performance of base-level learners. Thus, SM1 is adopted as the final predictor for the identification of DBPs in the subsequent analysis.

### 3.3. Comparison with Existing Methods

In this section, we evaluate the performance of PredDBP-Stack by performing the following two testing protocols for a fair comparison with the existing methods, including DNABinder [17], DNA-Prot [4], iDNA-Prot [28], iDNA-Prot|dis [5], Kmer1+ACC [14], iDNAPro-PseAAC [19], Local-DPP [27], HMMBinder [20], and StackDPPred [29].

The jackknife test is first implemented on the benchmark dataset PDB1075, and the detailed results are reported in Table 3. As shown in Table 3, HMMBinder, StackDPPred, and the proposed PredDBP-Stack provide outstanding performance with the OA higher than 85% and the AUC value more than 0.9. However, our method shows the best predictive power on the five metrics: OA (92.42%), SN (92.47%), SP (92.36%), MCC (0.85), and AUC (0.9677). This is likely attributable to the effective feature extraction technique from the HMM profile and the powerful stacked ensemble classifier adopted in the PredDBP-Stack model.

To further assess the robustness of the proposed method, we perform an independent test on the PDB186 dataset, where PredDBP-Stack is beforehand trained on the benchmark dataset.  Table 4 lists the predictive results of our method and nine existing state-of-the-art predictors mentioned above. From Table 4, we observe that our method, together with StackDPPred, performs better than the other methods on the PDB186 dataset, with the OA of 86.56%. Specifically, our method achieves the highest SP (86.02%) and AUC (0.8932) among the evaluated methods. In addition, the proposed PredDBP-Stack attains the second-best SN (87.10%) and MCC (0.731), which are slightly lower than those of StackDPPred. It should be pointed that the StackDPPred also applies a stacking technique to establish a powerful predictor for the identification of DBPs, which utilizes two different types of features, i.e., PSSM profile and residue wise contact energy profile [29]. However, our method also obtains favorable prediction accuracy when only the HMM profile is used. The successful applications of StackDPPred and PredDBP-Stack show that the stacking-based ML technique might yield a competitive tool for the prediction of DBPs and other protein classification tasks.

From the above comparisons, our method outperforms the existing models based on both the jackknife test and the independent test. This indicates that our method is a very promising tool for identifying DBPs and may at least play an important complementary role to existing methods.

## 4. Conclusions

Even though considerable efforts have been devoted so far, prediction of DBPs solely from sequence information still remains a challenging problem in bioinformatics. In this study, we develop a stacking-based ML model PredDBP-Stack to further improve prediction accuracy of DBPs, which employs an ensemble of base learners, such as KNN, LR, DT, and SVM, to generate outputs for the meta-classifier. Firstly, a hybrid feature encoding model, called AATP-HMM, is proposed to transform the HMM profiles to fixed-length numeric vectors, which incorporate evolution information and sequence-order effects. Next, these feature vectors are used to train the base-level predictors in the first stage. Then, GBDT is adopted as the meta-classifier in the second stage to implement the final prediction of DBPs. Finally, the jackknife cross validation and the independent test are performed on the two benchmark datasets to evaluate the predictive power of the proposed method. Comparison with the other existing predictors indicates that our method provides the outstanding improvement and could serve as a useful tool for predicting DBPs, given the sequence information alone.

## Figures and Tables

**Table 1:** Performance comparison of six base classifiers on the PDB1075 dataset using the jackknife test.

Method	OA (%)	SN (%)	SP (%)	MCC	AUC
DT	74.53	74.71	74.36	0.4906	0.7838
KNN	76.22	75.68	76.73	0.5240	0.8364
LR	78.18	78.19	78.18	0.5635	0.8508
XGB	78.74	75.64	80.69	0.5634	0.8624
RF	78.28	83.4	73.45	0.5702	0.8648
SVM	80.34	81.66	79.27	0.6091	0.8774

**Table 2:** Performance comparison of five SMs on the PDB1075 dataset using the jackknife test.

Method	OA (%)	SN (%)	SP (%)	MCC	AUC
SM1	92.42	92.47	92.36	0.8482	0.9677
SM2	92.23	91.89	92.55	0.8444	0.9664
SM3	91.76	91.31	92.18	0.8350	0.9635
SM4	79.87	82.82	77.09	0.5993	0.8745
SM5	90.54	90.93	90.18	0.8108	0.9560

**Table 3:** Performance comparison on the benchmark dataset PDB1075.

Method	OA (%)	SN (%)	SP (%)	MCC	AUC
DNA-Prot	72.55	82.67	59.76	0.44	0.7890
iDNA-Prot	75.40	83.81	64.73	0.50	0.7610
iDNA-Prot|dis	77.30	79.40	75.27	0.54	0.8260
DNABinder	73.95	68.57	79.09	0.48	0.8140
Kmerl+ACC	75.23	76.76	73.76	0.50	0.8280
iDNAPro-PseAAC	76.76	75.62	77.45	0.53	0.8392
Local-DPP	79.20	84.00	74.50	0.59	—
HMMBinder	86.33	87.07	85.55	0.72	0.9026
StackDPPred	89.96	91.12	88.80	0.80	0.9449
Our method	92.42	92.47	92.36	0.85	0.9677

**Table 4:** Performance comparison on the independent dataset PDB186.

Method	OA (%)	SN (%)	SP (%)	MCC	AUC
DNA-Prot	61.80	69.90	53.80	0.240	0.7960
iDNA-Prot	67.20	67.70	66.70	0.334	0.8330
iDNA-Prot|dis	72.00	79.50	64.50	0.445	0.7860
DNABinder	60.80	57.00	64.50	0.216	0.6070
Kmerl+ACC	70.96	82.79	59.13	0.431	0.7520
iDNAPro-PseAAC	69.89	77.41	62.37	0.402	0.7754
Local-DPP	79.00	92.50	65.60	0.625	—
HMMBinder	69.02	61.53	76.34	0.394	0.6324
StackDPPred	86.55	92.47	80.64	0.736	0.8878
Our method	86.56	87.10	86.02	0.731	0.8932

## Data Availability

The datasets and source codes for this study are freely available to the academic community at: https://github.com/taigangliu/PredDBP-Stack.

## References

[1] Langlois R. E., Lu H. (2010). Boosting the prediction and understanding of DNA-binding domains from sequence. *Nucleic Acids Research*.

[2] Qu K., Wei L., Zou Q. (2019). A review of DNA-binding proteins prediction methods. *Current Bioinformatics*.

[3] Lou W., Wang X., Chen F., Chen Y., Jiang B., Zhang H. (2014). Sequence based prediction of DNA-binding proteins based on hybrid feature selection using random forest and Gaussian Naïve Bayes. *PLoS ONE*.

[4] Kumar K. K., Pugalenthi G., Suganthan P. N. (2009). DNA-Prot: identification of DNA binding proteins from protein sequence information using random forest. *Journal of Biomolecular Structure & Dynamics*.

[5] Liu B., Xu J., Lan X. (2014). iDNA-Prot|dis: identifying DNA-binding proteins by incorporating amino acid distance-pairs and reduced alphabet profile into the general pseudo amino acid composition. *PLoS ONE*.

[6] Nimrod G., Szilágyi A., Leslie C., Ben-Tal N. (2009). Identification of DNA-binding proteins using structural, electrostatic and evolutionary features. *Journal of Molecular Biology*.

[7] Nimrod G., Schushan M., Szilágyi A., Leslie C., Ben-Tal N. (2010). iDBPs: a web server for the identification of DNA binding proteins. *Bioinformatics*.

[8] Wang Y., Ding Y., Guo F., Wei L., Tang J. (2017). Improved detection of DNA-binding proteins via compression technology on PSSM information. *PLoS ONE*.

[9] Motion G. B., Howden A. J. M., Huitema E., Jones S. (2015). DNA-binding protein prediction using plant specific support vector machines: validation and application of a new genome annotation tool. *Nucleic Acids Research*.

[10] Nanni L., Lumini A. (2008). Combing ontologies and dipeptide composition for predicting DNA-binding proteins. *Amino Acids*.

[11] Adilina S., Farid D. M., Shatabda S. (2019). Effective DNA binding protein prediction by using key features via Chou's general Pse AAC. *Journal of Theoretical Biology*.

[12] Rahman M. S., Shatabda S., Saha S., Kaykobad M., Rahman M. S. (2018). DPP-Pse AAC: a DNA-binding protein prediction model using Chou’s general Pse AAC. *Journal of Theoretical Biology*.

[13] Fu X., Zhu W., Liao B., Cai L., Peng L., Yang J. (2018). Improved DNA-binding protein identification by incorporating evolutionary information into the Chou’s Pse AAC. *IEEE Access*.

[14] Dong Q., Wang S., Wang K. Identification of DNA-binding proteins by auto-cross covariance transformation.

[15] Liu B., Wang S., Dong Q., Li S., Liu X. (2016). Identification of DNA-binding proteins by combining auto-cross covariance transformation and ensemble learning. *IEEE Transactions on Nanobioscience*.

[16] Liu B., Xu J., Fan S., Xu R., Zhou J., Wang X. (2015). Pse DNA-Pro: DNA-binding protein identification by combining Chou’s Pse AAC and physicochemical distance transformation. *Molecular Informatics*.

[17] Kumar M., Gromiha M. M., Raghava G. P. (2007). Identification of DNA-binding proteins using support vector machines and evolutionary profiles. *BMC Bioinformatics*.

[18] Waris M., Ahmad K., Kabir M., Hayat M. (2016). Identification of DNA binding proteins using evolutionary profiles position specific scoring matrix. *Neurocomputing*.

[19] Liu B., Wang S., Wang X. (2015). DNA binding protein identification by combining pseudo amino acid composition and profile-based protein representation. *Scientific Reports*.

[20] Zaman R., Chowdhury S. Y., Rashid M. A. (2017). HMMBinder: DNA-binding protein prediction using HMM profile based features. *Bio Med Research International*.

[21] Zhang J., Liu B. (2019). A review on the recent developments of sequence-based protein feature extraction methods. *Current Bioinformatics*.

[22] Zou Y., Ding Y., Tang J., Guo F., Peng L. (2019). FKRR-MVSF: a Fuzzy Kernel Ridge Regression Model for identifying DNA-binding proteins by multi-view sequence features via Chou's five-step rule. *International Journal of Molecular Sciences*.

[23] Ali F., Ahmed S., Swati Z. N. K., Akbar S. (2019). DP-BINDER: machine learning model for prediction of DNA-binding proteins by fusing evolutionary and physicochemical information. *Journal of Computer-Aided Molecular Design*.

[24] Nanni L., Brahnam S. (2019). Set of approaches based on 3D structure and position specific-scoring matrix for predicting DNA-binding proteins. *Bioinformatics*.

[25] Qu K., Han K., Wu S. (2017). Identification of DNA-binding proteins using mixed feature representation methods. *Molecules*.

[26] Zhang J., Liu B. (2017). PSFM-DBT: identifying DNA-binding proteins by combing position specific frequency matrix and distance-bigram transformation. *International Journal of Molecular Sciences*.

[27] Wei L., Tang J., Zou Q. (2017). Local-DPP: an improved DNA-binding protein prediction method by exploring local evolutionary information. *Information Sciences*.

[28] Lin W.-Z., Fang J.-A., Xiao X., Chou K. C. (2011). iDNA-Prot: identification of DNA binding proteins using random forest with grey model. *PLoS ONE*.

[29] Mishra A., Pokhrel P., Hoque M. T. (2019). Stack DPPred: a stacking based prediction of DNA-binding protein from sequence. *Bioinformatics*.

[30] Liu X.-J., Gong X.-J., Yu H. (2018). A model stacking framework for identifying DNA binding proteins by orchestrating multi-view features and classifiers. *Genes*.

[31] You W., Yang Z., Guo G., Wan X. F., Ji G. (2019). Prediction of DNA-binding proteins by interaction fusion feature representation and selective ensemble. *Knowledge-Based Systems*.

[32] Qu Y.-H., Yu H., Gong X.-J., Xu J. H., Lee H. S. (2017). On the prediction of DNA-binding proteins only from primary sequences: a deep learning approach. *PLoS ONE*.

[33] Chauhan S., Ahmad S. (2020). Enabling full-length evolutionary profiles based deep convolutional neural network for predicting DNA-binding proteins from sequence. *Proteins*.

[34] Hu S., Ma R., Wang H. (2019). An improved deep learning method for predicting DNA-binding proteins based on contextual features in amino acid sequences. *PLoS ONE*.

[35] Wolpert D. H. (1992). Stacked generalization. *Neural Networks*.

[36] Xiong Y., Wang Q., Yang J., Zhu X., Wei D. Q. (2018). Pred T4SE-Stack: prediction of bacterial type IV secreted effectors from protein sequences using a stacked ensemble method. *Frontiers in Microbiology*.

[37] Berman H. M., Westbrook J., Feng Z. (2000). The protein data Bank. *Nucleic Acids Research*.

[38] Chen J., Long R., Wang X. L., Liu B., Chou K. C. (2016). dRHP-Pse RA: detecting remote homology proteins using profile-based pseudo protein sequence and rank aggregation. *Scientific Reports*.

[39] Lyons J., Paliwal K. K., Dehzangi A., Heffernan R., Tsunoda T., Sharma A. (2016). Protein fold recognition using HMM-HMM alignment and dynamic programming. *Journal of Theoretical Biology*.

[40] Remmert M., Biegert A., Hauser A., Söding J. (2012). HHblits: lightning-fast iterative protein sequence searching by HMM-HMM alignment. *Nature Methods*.

[41] Consortium T. U. (2017). Uni Prot: the universal protein knowledgebase. *Nucleic Acids Research*.

[42] Cortes C., Vapnik V. (1995). Support-vector networks. *Machine Learning*.

[43] Cover T., Hart P. (1967). Nearest neighbor pattern classification. *IEEE Transactions on Information Theory*.

[44] Hosmer D. W., Lemeshow S., Sturdivant R. X. (2013). *Applied logistic regression*.

[45] Tin Kam H. Random decision forests.

[46] Safavian S. R., Landgrebe D. (1991). A survey of decision tree classifier methodology. *IEEE Transactions on Systems, Man, and Cybernetics*.

[47] Chen T., Guestrin C. XGBoost: a scalable tree boosting system.

[48] Pedregosa F., Varoquaux G., Gramfort A. (2011). Scikit-learn: machine learning in Python. *Journal of Machine Learning Research*.

[49] Friedman J. H. (2001). machine. *The Annals of Statistics*.

[50] Friedman J. H. (2002). Stochastic gradient boosting. *Computational Statistics & Data Analysis*.

[51] Lv Z., Jin S., Ding H., Zou Q. (2019). A random forest sub-Golgi protein classifier optimized via dipeptide and amino acid composition features. *Frontiers in Bioengineering and Biotechnology*.

[52] Meng C., Wei L., Zou Q. (2019). Sec Pro MTB: support vector machine-based classifier for secretory proteins using imbalanced data sets applied to mycobacterium tuberculosis. *Proteomics*.

[53] Meng C., Jin S., Wang L., Guo F., Zou Q. (2019). AOPs-SVM: a sequence-based classifier of antioxidant proteins using a support vector machine. *Frontiers in Bioengineering and Biotechnology*.

[54] Lv Z., Zhang J., Ding H., Zou Q. (2020). RF-Pse U: a random forest predictor for RNA pseudouridine sites. *Frontiers in Bioengineering and Biotechnology*.

